# Is there Emergence of Clinical HBV Resistance Under Long-Term HBV Combination Therapy? A Challenging Case Report

**DOI:** 10.3390/v2081564

**Published:** 2010-07-29

**Authors:** Knud Schewe, Christian Noah, Hüseyin Sirma, Stefan Schmiedel, Jan van Lunzen, Jürgen Kurt Rockstroh, Oliver Schildgen

**Affiliations:** 1 Infektionsmedizinisches Centrum Hamburg (ICH St. Georg), Germany; E-Mail: schewe@ich-hamburg.de; 2 IPM Biotech GmbH, Labor Lademannbogen, Hamburg, Germany; E-Mail: christian.noah@labor-lademannbogen.de; 3 Heinrich-Pette-Institute, Hamburg, Germany; E-Mail: sirhus@rocketmail.com; 4 University Hospital Eppendorf (UKE), Hamburg, Germany; E-Mails: stefan.schmiedel@uke-hh.de (S.S.); v.lunzen@uke.uni-hamburg.de (J.v.L.); 5 University Hospital Bonn, Germany; E-Mail: Juergen.Rockstroh@ukb.uni-bonn.de; 6 Kliniken der Stadt Köln gGmbH, Cologne, Germany

**Keywords:** HIV-HBV-coinfection, tenofovir, clinical HBV resistance

## Abstract

A first case of clinical tenofovir (TDF) HBV resistance in an HIV/HBV coinfected patient who developed an acute flare of hepatitis B is reported. The clinical course was accompanied by signs of acute liver failure after being on successful HBV treatment with tenofovir and persistently undetectable HBV-DNA viral load for over five years.

## Case Report

We report the case of a 44-year-old HIV/HBV co-infected homosexual patient who was on HBV treatment with tenofovir for over five years with HBV virus load below the detection limit. In August 2008 he developed an acute flare of hepatitis B with signs of acute liver failure. All subsequent procedures described below were performed after written informed consent and in accordance with a positive vote by the local ethical committees.

Chronic HBeAg-positive HBV-infection was diagnosed in 1995. He was treated with non-modified interferon (5×10^7^U; 3×/week) for 12 months, without any response. In 1997 HIV infection was diagnosed with a CD4 Nadir of 280/μl. Antiretroviral therapy (ART) was initiated in 1997 combining zidovudine (AZT), lamivudine (3TC) and indinavir (IDV). In October 1999 the patient was referred to our clinic.

Determination of HBV-DNA load revealed approximately >6×log10 genome equivalents and an HIV load of 4×log10 copies/ml, with the GPT/ALT being 45 U/L and CD4 600/μl. HBV sequence analysis was not performed at that time. Genotypic HIV sequencing revealed the rt-mutations M184V, L214F, L90M. HIV therapy was subsequently switched to abacavir (ABC), didanosine (DDI), stavudine (d4T) and 3TC leading to a drop of HIV titer below 50 copies/ml.

**Figure 1.**
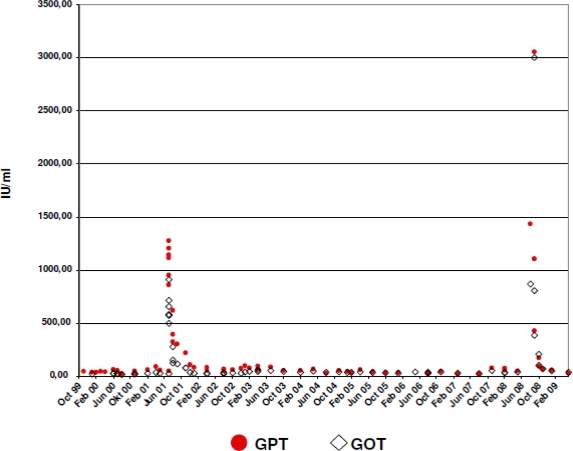
Fluctuation of liver enzymes throughout the whole observation period (IU/ml). GPT = ALT, GOT = AST.

**Figure 2.**
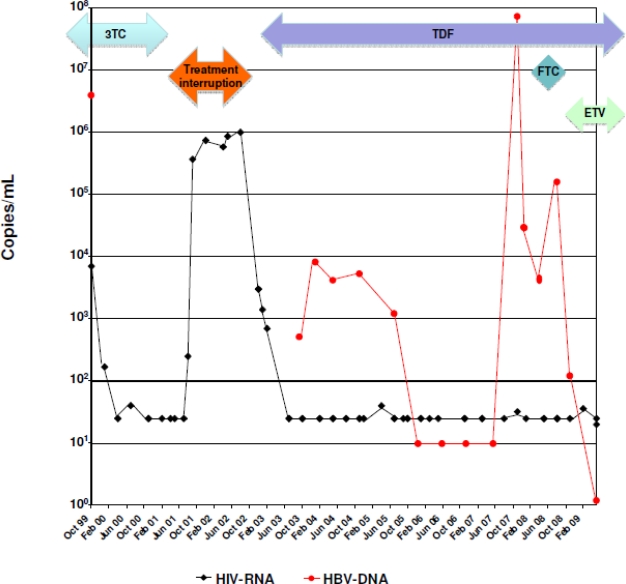
Course of viremia of both HIV-RNA and HBV-DNA (genome copies per ml plasma) throughout the observation period. 3TC: Lamivudine; TI: Treatment Interruption; TDF: Tenofovir; FTC: Emtricitabine; ETV: Entecavir.

An acute toxic hepatitis (Figure 1) due to abuse of anabolic steroids necessitated an interruption of ART in 06/2001 and resulted in an increase of HIV viral load (Figure 2). In 09/2002 ART was resumed with the combination of tenofovir, DDI, ABC and efavirenz (EFV). During the next years, HBV-titer declined below the detection level (04/2003: <1000 copies/ml; 10/2003: 8000c/ml; 02/2004: 4000c/ml; 08/2004: 5000c/ml; 04/2005: 1000c/ml; 10/2005: <100c/ml; 11/2006: <70c/ml; 03/2007: <70c/ml).

In 2004 hypertension was diagnosed and treated with ramipril and hydrochlorothiazide. Acute myocardial infarction with cardiac arrest and emergency stenting of the left main coronary artery occurred in January 2008. Coronary artery disease and cardiac insufficiency with an ejection fraction of 25% was treated with clopidogrel, acetyl salicylic acid (ASS), carvedilol, ramipril, torasemid and simvastatin. In response to the publication of D:A:D cohort data showing an increased risk of myocardial infarction in patients treated with DDI and ABC, the latter drugs were discontinued in June 2008 and the ART regimen was switched to Emtricitabine (FTC), AZT, TDF and EFV [1]. The patient did not show up for scheduled laboratory examinations in 07/2008. In 08/2008 clinically severe acute hepatitis with signs of liver failure developed (ALT 3.060 U/l, AST 3004 U/l, AP 189 U/l, Bilirubin 24 mg/dl, INR 1,59, and *(Pseudo-)Cholinesterase*/CHE 4060 U/l). At this time HBV-load increased to 200.000 IU/ml (approx. 1×10^7^c/mL). Abuse of illicit drugs, herbal medications or anabolic steroids was not recorded. Autoimmune hepatitis, Wilson’s disease, Hemochromatosis, acute viral hepatitis A/C/D/E were excluded by laboratory examinations. The patient was admitted to the hospital for impending liver failure. Liver biopsy revealed expanded lymphocytic portal-lobular inflammation with necrosis and apoptotic areas, multifocal border zone activity, portal fibrosis and 30% fatty parenchyma that was suspected to have a toxic component. Concerns of possible drug toxicity led to the discontinuation of simvastatin, clopidogrel, and FTC. Meanwhile Entecavir was added to the regimen and led to a rapid decline of HBV viremia, improvement of liver function tests and clinical symptoms (Figure 1). HBV-DNA became undetectable in 01/2009 and transaminases returned to normal levels in 05/2009. Analysis of stored frozen plasma samples revealed HBV titer of 9.800.000 U/ml in 08/2007, 20.000 U/ml in 11/2007 and 4.200 U/ml in 05/2008. In 11/2007 serum transaminases were elevated for the first time since 04/2005 (ALT: 80 U/l, AST: 53 U/l).

At the time of the flare in 08/2008 sequencing of the HBV polymerase was performed in three independent laboratories with identical results. The patient is infected with an HBV genotype A (99.8% similarity). Although the patient was continuously treated with TDF+FTC, no mutations known or suspected to contribute to TDF/3TC/FTC resistance were found except polymorphisms rtL217R and rtS219A. However, none of them are mediating or associated with TDF resistance [2,3,4].

Despite the lack of mutations leading to tenofovir (TDF) resistance, the patient suffered from clinical resistance to anti-HBV therapy. HBV DNA levels fluctuated strongly despite continuous treatment with TDF over one year with minimal increases in transaminases (1–2 times the upper limit of normal). Analysis of 165 HIV/HBV-coinfected patients in France showed that clinical resistance to tenofovir occurred only in two cases, both of them infected with HBV genotype A [5]. Here, previously undescribed R274W and S219A mutations were found, but no A194T mutation was detected. As a matter of speculation, however, those mutations or hitherto un-observed mutations located in the periphery of the putative active domain of the viral reverse transcriptase may be responsible for the virological breakthrough, highlighting the need for systematic analyses of the whole viral polymerase gene in case of resistance [1–17]. As long as no crystal structure of the molecule exists computer assisted models may be a good approximation in the prediction of the resistance profile when a given mutation occurs.

The HIV load during the HBV flare remained below 50 copies/ml, thus clearly proving the patients’ adherence to antiviral therapy particularly accounting for the low genetic barrier of an efavirenz based antiretroviral regimen. Nevertheless, although general lack of treatment adherence is most unlikely in view of HIV viremia remaining suppressed, it cannot be fully ruled out that the patient was not fully adherent to TDF. In this context extremely high HBV load of 9.800.000 U/ml in 08/2007 is intriguing and a measurement of the TDF drugs levels should have occurred to fully exclude any lack of compliance. Since some rt217R variant HBV-strains remain susceptible to antiviral therapy, it is rather unlikely that this mutation alone accounts for the observed clinical resistance. It appears likely that resistance to antiviral therapy in HBV infected patients is due to – so far unknown - host mechanisms [17]. In conjunction with pre-existing liver damage components of the complex antiretroviral and cardiovascular drug regimen may have contributed to liver dysfunction which in turn led to the clinical picture of acute liver failure in our patient.
